# Ventral mesh rectopexy for recurrent rectal prolapse after Altemeier perineal proctosigmoidectomy: feasibility and outcomes

**DOI:** 10.1007/s00423-024-03227-w

**Published:** 2024-02-02

**Authors:** Lukas Schabl, Tracy Hull, Kamil Erozkan, Ali Alipouriani, Kristen A. Ban, Scott R. Steele, Anna R. Spivak

**Affiliations:** Department for Colorectal Surgery, Digestive Disease Institute, Cleveland Clinic, 9500 Euclid Ave./A 30, Cleveland, OH 44195 USA

**Keywords:** Altemeier, Proctosigmoidectomy, Rectal prolapse, Ventral mesh rectopexy, Recurrence

## Abstract

**Purpose:**

Recurrence of rectal prolapse following the Altemeier procedure is reported with rates up to 40%. The optimal surgical management of recurrences has limited data available. Ventral mesh rectopexy (VMR) is a favored procedure for primary rectal prolapse, but its role in managing recurrences after Altemeier is unclear. VMR for recurrent prolapse involves implanting the mesh on the colon, which has a thinner wall, more active peristalsis, no mesorectum, less peritoneum available for covering the mesh, and potential diverticula. These factors can affect mesh-related complications such as erosion, migration, or infection. This study assessed the feasibility and perioperative outcomes of VMR for recurrent rectal prolapse after the Altemeier procedure.

**Methods:**

We queried our prospectively maintained database between 01/01/2008 and 06/30/2022 for patients who had experienced a recurrence of full-thickness rectal prolapse following Altemeier’s perineal proctosigmoidectomy and subsequently underwent ventral mesh rectopexy.

**Results:**

Ten women with a median age of 67 years (range 61) and a median BMI of 27.8 kg/m^2^ (range 9) were included. Five (50%) had only one Altemeier, and five (50%) had multiple rectal prolapse surgeries, including Altemeier before VMR. No mesh-related complications occurred during a 65-month (range 165) median follow-up period. Three patients (30%) experienced minor postoperative complications unrelated to the mesh. Long-term complications were chronic abdominal pain and incisional hernia in one patient, respectively. One out of five (20%) patients with only one previous prolapse repair had a recurrence, while all patients (100%) with multiple prior repairs recurred.

**Conclusion:**

Mesh implantation on the colon is possible without adverse reactions. However, high recurrence rates in patients with multiple previous surgeries raise doubts about using VMR for secondary or tertiary recurrences.

## Introduction

Rectal prolapse is the protrusion of one or all layers of the rectum through the anus [1]. The treatment for rectal prolapse is surgical and can be performed via perineal and abdominal approaches. Abdominal approaches include suture rectopexy, ventral mesh rectopexy, and resection rectopexy [2]. Abdominal approaches are favored as initial procedures in healthy and younger patients [1, 2]. The American Society of Colon and Rectum Surgeons (ASCRS) recommend perineal techniques in frail patients, such as Delorme and Altemeier procedure. This is argued with a decreased surgery time, repeatability, and the possibility of performing without general anesthesia and pneumoperitoneum [2]. The ASCRS guidelines reflect the current practice and understanding of abdominal and perineal approaches, even though studies from the last two decades have reported conflicting results comparing perineal and abdominal techniques [1]. The primary Altemeier procedure presents a recurrence rate of 0–58% with varying follow-up periods [1, 3]. The recurrence rate necessitates surgical options for redo procedures. Redo procedures can be performed either via perineal or abdominal approach. Abdominal mesh rectopexy is the preferred procedure in otherwise healthy patients with a first presentation of rectal prolapse [2]. It can be considered in recurrence after a perineal procedure if the patient’s risk profile permits the approach [1, 3, 4]. After a previously performed Altemeier procedure with proctectomy, the mesh needs to be anchored on the colon. Colonic mesh applications lack scientific reports and require improved understanding. We hypothesized that mesh implanted on the colon does not cause mesh-related complications and is feasible with an improved rate of recurrence.

## Methods

Using an institutional review board-approved prospectively maintained rectal prolapse database, we identified patients with rectal prolapse recurrence after the Altemeier procedure and subsequent repeat surgery with ventral mesh implantation. Patients were deemed fit enough to safely undergo an abdominal procedure. Patients who underwent treatment for rectal prolapse were followed to screen for complications, recurrences, and subjective treatment success. Each patient was contacted individually to complete a structured questionnaire focused on the remaining symptoms and quality of life.

The extracted data included demographics (age, sex, BMI, and ASA) and medical history (indication for the Altemeier procedure, previous surgeries, obstetrical history, and outcomes (recurrence, postoperative, and long-term complications)). To verify the comparability of the surgeries, each operative report was reviewed. During the initial Altemeier procedure, all patients underwent proctosigmoidectomy, resection of the prolapsed rectum, and coloanal anastomosis.

**Fig. 1:**
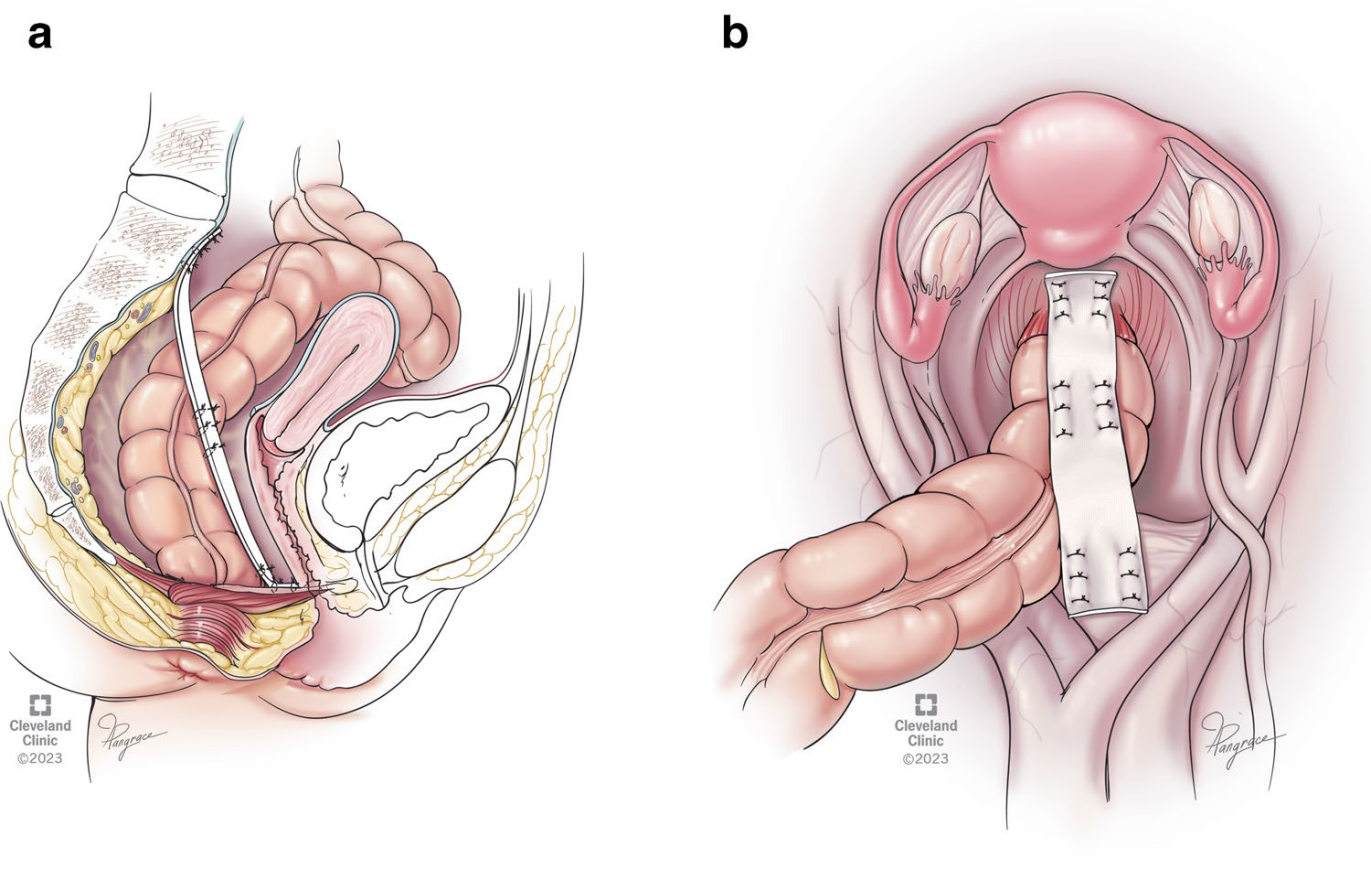
Anatomical scheme for ventral mesh rectopexy after Altemeier procedure, **A** sagittal plane and **B** transverse plane

In ventral mesh rectopexy, prolapse repair is performed by dissection of the rectovaginal septum, followed by mesh fixation to the anterior rectum and sacral promontory, followed by reperitonealization of the mesh. The rectum is the usual site for mesh implantation in ventral mesh rectopexy. With the rectum resected after the Altemeier procedure, the mesh is anchored to the colon, not the rectum (Fig. 1A and B). In this study, we analyzed postoperative complications as well as short- and long-term outcomes to determine a possible adverse reaction of the colon to the mesh as a foreign body and therefore safety and feasibility of the procedure. Long-term recurrence, fecal incontinence, and patient satisfaction are also reported. Continuous data were reported as medians with ranges and categorical data as proportions (%).

## Results

### Patients’ demographics

Ten patients with previous perineal proctosigmoidectomies treated with ventral mesh rectopexy for rectal prolapse recurrences were identified between 01/01/2008 and 06/30/2022. All patients were women. The median age was 67 years (range 34–95); the median body mass index was 27.8 kg/m^2^ (range 23.6–32.6), and the median American Society of Anesthesiology score was 3 (range 2–3) at the time of surgery.

### Surgical history

The initial Altemeier procedure was performed due to full-thickness rectal prolapse in all the patients. The reasons for choosing the Altemeier procedure in the first place were available for six patients. The reasons were a recent appendectomy for phlegmonous appendicitis, current ileostomy, a surgeon’s recommendation, a combined perineal procedure with total colpectomy and perineoraphy, the patient’s age, and a previously performed Delorme procedure in one patient.

Before mesh implantation, five patients were treated with other procedures for rectal prolapse in addition to the Altemeier procedure. Two patients underwent an additional Altemeier procedure and suture rectopexy; two patients underwent a single additional Altemeier procedure, and one patient underwent an additional Delorme procedure (Table 1). Other previous pelvic surgeries included hysterectomies in seven patients and bladder suspensions in two.

**Table 1:** Patients treated with different procedures

Age at VMR (years)	Gender	Previous surgeries	Mesh type	Time to recurrence (months)	Surgical therapy	Follow-up (years)	Quality of life
89	F	1 Altemeier; Delorme	Synthetic	13	Altemeier	5	-
67	F	2 Altemeier; suture rectopexy	Synthetic	11	Altemeier and loop-ileostomy	8	Worsened
75	F	2 Altemeier	Biological	37	Suture rectopexy	6	-
63	F	2 Altemeier; suture rectopexy	Synthetic	7	Proctectomy and end-colostomy	7	-
89	F	2 Altemeier	Synthetic	25	Suture rectopexy	21	-
95	F	1 Altemeier	Synthetic	2	-	2	-
60	F	1 Altemeier	Biological	-	-	7	Improved
63	F	1 Altemeier	Synthetic	-	-	2	Improved
34	F	1 Altemeier	Biological	-	-	14	-
77	F	1 Altemeier	Synthetic	-	-	4	Improved

*VMR* ventral mesh rectopexy, *F* Female

Nine patients had combined 18 childbirths. Data were available for nine births, all of which were vaginal deliveries.

### Mesh implantation for recurrence after Altemeier procedure

All patients were treated with ventral mesh rectopexy between 2008 and 2022. In seven (70%) patients, the surgery was performed robotically, in two (20%) patients laparoscopically, and in one (10%) patient open. Three (30%) patients underwent combined pelvic floor surgery, including two sacro-colpoperineopexies and one sacro-hystero-colpoperineopexy with a tension-free vaginal obturator sling procedure.

A biological mesh was used in three (30%) patients. The thickness was tailored to be 6ply in two patients and 4ply in one patient. The remaining seven (70%) patients were treated with synthetic mesh. Reasons why VMR was performed were available in nine (90%) patients. VMR was argued in five (55.5%) patients with two previously performed perineal procedures, in one (11.1%) with a simultaneous laparotomy for repair of a large ventral hernia, in one (11.1%) patient with combined surgery for pelvic organ prolapse, and in one (11.1%) patient with a previously failed perineal approach and current fecal incontinence. One (11.1%) patient specifically requested a transabdominal approach.

### Outcomes after ventral mesh rectopexy

The median follow-up period was 65 months (range 2–167). Postoperative complications during the first 30 days occurred in three (30%) patients and involved one atrial fibrillation without the need for intervention, one temporary postoperative delirium, and one urinary retention. Long-term complications, excluding recurrences, involved one incisional hernia and chronic abdominal pain in one patient. Fecal incontinence persisted in five patients.

During the follow-up period, six (60%) patients reported recurrence after the ventral mesh rectopexy after a median time to recurrence of 12 months (range 2–37). Five (80%) patients presented with recurrent full-thickness rectal prolapse and one (20%) with mucosal rectal prolapse, of whom five patients were treated surgically. One patient underwent a new Altemeier procedure, one a proctectomy with end-colostomy with combined mesh removal, and another one an Altemeier procedure with temporary loop-ileostomy. Two patients were treated with suture rectopexy and one patient was treated conservatively (Table 1).

Quality of life assessment was available for three out of four patients (75%) with no recurrence, who reported improved quality of life. Quality of life assessment was available in one out of six (16.7%) with recurrence. This patient reported a worsened quality of life (Table 1).

## Discussion

Full-thickness rectal prolapse is a condition for which the optimal surgical treatment has not yet been found, as shown by numerous possible approaches, techniques, modifications, and uncertainty about mesh implantation [2]. The geographical preferences for surgical techniques further underscore the ongoing search [4]. Less common in Europe and Asia, approximately 60% of patients with full-thickness rectal prolapse in the USA undergo a perineal procedure, of which Altemeier is the most common [5]. Due to recurrence rates after Altemeier procedure being as high as 58%, colorectal surgeons have to treat patients with recurrent prolapse after previous proctectomy [3]. Table 2 provides a summary of the literature reporting recurrence rates after the Altemeier procedure.

**Table 2:** Summary of literature reporting the recurrence rate after Altemeier procedure

Study	Patients	Rate of recurrence	Follow-up (months)
Porter N. H. (1971)	110	58%	10
Friedman R. et al. (1983)	27	35%	12–204
Gopal K. A. et al. (1984)	18	6%	12
Prasard M. L. et al. (1986)	25	0	36
Ramanujam PS et al. (1994)	41	5.5%	48
Kim D. S. et al. (1999)	183	16%	64
Kimmins M. H. et al. (2001)	63	6.4%	20.8
Sobrado C. W. et al. (2004)	12	0	49
Chun S. W. et al. (2004)	109	16.5%	28.8
Habr-Gama et al. (2006)	44	7.1%	49
Glasgow S. C. et al. (2008)	103	8.5%	36
Altomare D. F. et al. (2008)	93	18%	41
Kim M. et al. (2010)	38	3%	24
Cirocco W. et al. (2010)	103	0	43
Riansuwan W. et al. (2010)	55	26.5%	37
Ozawa S. et al. (2010)	13	7.6%	29.3
Lee S. H. et al. (2011)	123	11.3%	12.8
Ding J. H. et al. (2012)	113	17.7%	42
Ris F. et al. (2012)	60	13.3%	48
Senapati A. et al. (2013)	102	23.5%	36
Tiengtianthum R. et al. (2014)	518	22.6%	16.2
Elagili F. et al. (2015)	22	9%	13
Pinheiro L. V. et al. (2016)	33	26.7%	50
Trompetto M. et al. (2019)	43	Est. 40%	49
Boccasanta P. et al. (2021)	110	24.6%	89

Surgical options for recurrence are numerous, the same as initial rectal prolapse options, and reflect the need for further studies. The lack of evidence is also made obvious by a missing recommendation regarding the treatment for recurrent rectal prolapse in the current ASCRS guidelines [2]. Pikarsky et al. reported the same recommendations for initial rectal prolapse in patients with recurrent rectal prolapse [6]. Steele et al. suggested abdominal repair for recurrent rectal prolapse if the patient’s risk profile is suitable based on an 8% risk for a second recurrence after abdominal repair compared to 50% after perineal repair [7].

One of the reasons why the Altemeier procedure is advocated in the elderly and frail patient population is its repeatability [6, 8, 9]. However, redo Altemeier procedure might come at the price of an even higher recurrence rate [7, 8]. Abdominal procedures are preferred in eligible patients with initial rectal prolapse as lower recurrence rate is well documented in multiple studies [1, 7, 10]. While this recommendation has found its way into guidelines [2], other studies report contradicting results on the recurrence rate of abdominal and perineal procedures [4, 11–13]. Patients with an improved risk profile might become eligible for abdominal repair for the recurrence [7]. Ventral mesh rectopexy can be offered in this patient group, defined as recurrent rectal prolapse after the previous Altemeier procedure. While primary and recurrent prolapse has been treated with a mesh repair, the effect of mesh implantation on the colon at ventral mesh rectopexy after the Altemeier procedure has not been investigated.

To date, only one other study has specifically reported mesh implantation after the Altemeier procedure [14]. Atallah et al. attempted to decrease the recurrence rate of the Altemeier procedure by combining the procedure with perineal biological mesh implantation; they used biologic mesh to buttress pelvic muscles to create additional support at the time of the repair. The follow-up period was limited to 6 months, and no complications or prolapse recurrences were observed [14].

In our study, six out of ten patients (60%) presented with recurrent prolapse after a median of 12 months, with five needing additional surgery. The longer time to recurrence in our study might explain why Atallah et al. did not report any recurrence. Additionally, all our patients with recurrence had previous prolapse surgeries, whereas only two patients in Atallah et al.’s cohort had undergone a previous Altemeier procedure [14]. Five out of six patients in our cohort with recurrence after ventral mesh rectopexy had multiple previous surgeries for rectal prolapse. The recurrence rate for ventral mesh rectopexy without previous rectal prolapse surgery varies from 3.7 to 15.4% [15–18]. The risk for a second recurrence is higher than the risk for first recurrence [7, 8]. Steele et al. reported the risk for a second recurrence after an abdominal approach at 8%. However, the authors did not clarify whether a mesh was implanted during the abdominal procedure or the technique used for the first prolapse surgery [7]. A multicenter retrospective study reported a second recurrence rate of 23.3% after any perineal technique as the initial procedure and any abdominal technique as the second [19]. Our recurrence rate after ventral mesh rectopexy is high but mainly affects patients with multiple previous surgeries for rectal prolapses (100%). In patients who underwent only one previous Altemeier procedure, only one recurrence (20%) occurred.

Mesh-related complications are unknown for meshes implanted on the colon. Mesh-related complications after ventral mesh rectopexy without previous Altemeier are low at 0–2.4% for synthetic mesh and 0–0.7% for biologic mesh [20]. Possible mesh-related complications after rectopexy include mesh erosion of the bowel or vaginal wall, mesh migration, and mesh infection, but are generally rare [18, 21–23]. Mesh-related complications to the colon are reported for intraperitoneal mesh, particularly after hernia repair [24].

In our study, no mesh-related complications occurred during the median follow-up period of 65 months after the ventral mesh rectopexy. Although our patient number was low, our long-term data showed no adverse colonic reaction to the mesh as a foreign body.

Patients treated with the Altemeier procedure are often older and frailer than those treated with abdominal procedures. The median age of our patients was higher than that of previously reported cohorts that underwent ventral mesh rectopexies [1, 7, 12]. The increased age in our study might be explained by ventral mesh rectopexy being the surgical treatment of recurrence. Therefore, patients have become older since the initial surgery. Our data are consistent with previous reports showing predominantly elderly women diagnosed with rectal prolapse [1, 2]. Eight of the ten women had a history of vaginal delivery [11]. We conclude that our cohort reflects the general population treated for rectal prolapse and that the results might reflect similar outcomes in a larger population.

Minimally invasive surgery has clear advantages over open surgery in terms of postoperative recovery and complication rates. The narrow anatomy of the pelvis can compromise visibility during open surgery [25]. These advantages are substantiated by the growing utilization of laparoscopy and robotics in the field. In fact, in 2019, robotics were employed in 41% of all rectopexies performed in Australia [26]. Moreover, the ASCRS young surgeons committee conducted a questionnaire survey that reported a significant 63% usage of robotic-assisted surgery in rectopexies [27]. Our results align with these findings, revealing a 70% utilization of robotics in ventral mesh rectopexies. Notably, a recent meta-analysis comparing laparoscopic and robotic ventral mesh rectopexy concluded that both approaches yielded comparable outcomes, with the added benefit of a shorter hospital stay in robotics [25].

Fifty to seventy-five percent of patients with rectal prolapse also suffer from fecal incontinence. Resection procedures are not advocated in patients with incontinence because the resolution of fecal incontinence appears to be lower when sigmoid resection is performed [2]. Improvement of fecal incontinence has been reported in 20–60% of patients for ventral mesh rectopexies [23]. In our study, five patients (50%) had persistent fecal incontinence after ventral mesh rectopexies. Previous proctosigmoidectomy may explain the limited improvement in these patients.

The percentage of women aged > 50 years who underwent hysterectomy in 2018 was 31.7% [28]. Interestingly, our study found that eight of ten patients had a history of hysterectomy. Barham et al. suggest an increased risk of combined genital and rectal prolapse after hysterectomy but state the incidence is unknown [29]. While hysterectomy might weaken the pelvic floor by removing supporting pelvic ligaments and nerve supply, no study has directly defined hysterectomies as a risk factor for rectal prolapse [30]. Hysterectomy is a major pelvic surgery that alters pelvic anatomy in women. Previous pelvic surgeries might increase the risk of recurrence of rectal prolapse, similar to how previous rectal prolapse surgeries increase the risk of re-recurrence. We point out that the number of patients in our study was too small to draw definitive conclusions.

Ventral mesh rectopexies after the Altemeier procedure were consistently performed using the same technique. First, the abdominal cavity was accessed, and the sacral promontory was cleared for future mesh fixation. The peritoneum was further opened to the bottom of the pelvic floor while both ureters were identified. Two EEA sizers were used to facilitate vaginal and neo rectal retraction to help identify the anatomy. All adhesions were lysed, freeing the colonic conduit circumferentially. The mesh was then anchored to the sacral promontory, levator ani muscles, and colonic conduit wall and fully reperitonialized. The anchor points of the mesh in VMR after Altemeier procedure are comparable to the ones of VMR for primary rectal prolapse. However, potential colonic diverticulosis has to be noted as well as possible difficulties in fully reperitonializing the mesh. The pelvic floor muscles might be thinned out and difficult to distinguish in cases of multiple previous prolapse repairs.

The procedure was tailored to patients who had undergone previous abdominal surgery. The technical steps of ventral mesh rectopexy after the Altemeier procedure are very similar to those of ventral mesh rectopexy without previous perineal rectal prolapse surgeries [2, 17]. We think the comparability of the procedures increases the comparability of patient outcomes.

This study was limited by its retrospective nature and the small sample size. The small sample size identified from a tertiary center over a 14-year period reflects the rarity of mesh implantation after the Altemeier procedure. Potentially, small numbers also reflect surgeons’ hesitation about mesh implantation on the colon. Due to the small sample size, no generalization about safety can be made. Analysis of quality of life was limited by the missing data of six patients. However, decreased satisfaction in four patients was likely because they all experienced recurrent prolapses.

## Conclusion

Ventral colonic mesh implantation after the Altemeier procedure is associated with a high recurrence rate, particularly in patients with a complicated history of multiple rectal prolapse recurrences and previous surgical procedures. In this small patient sample, no adverse reactions to the mesh implanted on the colon occurred. VMR after Altemeier procedure should not be routinely applied but reserved for a specific patient subset treated by experts in centers of excellence. When counseling patients for VMR after Altemeier procedure, the high recurrence rate and potential complications must be discussed. Studies with more patients are needed to draw further and more secure conclusions.

## Data Availability

The data, code, and other materials are available from the corresponding author upon request.

## Abbreviations

ASCRS: American Society of Colon and Rectum Surgeons
VMR: Ventral mesh rectopexy

## Declarations

### Meeting presentation

This study was accepted for presentation at the annual meeting of the American Society of Colon & Rectal Surgeons, June 1–4, 2024, Baltimore, MD, USA.
